# Report of the Diseases of Edinburgh for August, 1809

**Published:** 1809-10

**Authors:** John Roberton


### *Report of the Diseases of Edinburgh for August,* 1809.

By JOHN ROBERTON, M. D.

We have not, within the recollection of the oldest persons among us, had, at the same period of the year, so much, and such heavy falls of rain as has happened during the present month. Besides, the thunder and lightning, especially during the first half of the month, was excessive; not only, as is most customary, during the day, but often for whole nights together; the flashes, accompanied by the most tremendous peals of thunder, were uncommonly vivid.

For several days near the beginning of the month, we annually annually have some heavy falls of rain, known by the name of *Lammas* floods; but this season, as just observed, has far surpassed any former one, and consequently has been injurious to the country in various respects. About the beginning of the last third of the month, however, the rain was less constant, though, when it did occur, it was very heavy; and, about this time, especially towards evening, there were easterly winds which enveloped the city in fog. The month terminated with one or two good days.

The barometer, though low, did not sink so far as, from the heavy falls of rain, might have been expected.

The thermometer, for the most part, stood about 60°: it was indeed sometimes higher, but that was about its general range.

Fevers of a bad kind, catarrh, cynanche tonsillaris of a very bad kind, rheumatism, and bowel complaints, have been the most prevalent of the month.

The cases of fever have become of a more contagious and malignant nature than during the whole previous months of summer. In some numerous but poor families, it is not uncommon to find the greater part of them confined to bed by such fevers, and in many instances they have proved fatal, independently of the greatest care and attention that could be bestowed upon them. As noticed on a former occasion, I uniformly order the poorer sufferers from this disease to be instantly removed to the Royal Infirmary, and those differently situated to be carried to the highest and best aired rooms in the house. These means, even indepeudently of medicines, have always been attended with the happiest effects. This, itself, points out a principle, had we thought enough to adopt it, which at all times would most certainly lessen the severity, if not put a total stop to the rapid propagation of such diseases.

Catarrh still continues to rage with unabated violence: indeed, it of late seems to have increased in every respect, both in the number affected, and in its severity. This is evidently owing to the dampness of the weather, and to our exposure to the fogs after sun-set. In some habits, I have found the removal of the symptoms of this complaint extremely tedious, especially in consumptive habits. Their removal, however, in general, was effected by confinement within doors for a few days, and by cathartic medicines, occasional sudorifics, and the habit of bathing the feet in hot water immediately before going to bed.

Cynanche tonsillaris, though not universally prevalent in in the city, has appeared in various parts of it in a very bad form. Although it has principally been met with among infants and children, yet it has also appeared in those more advanced in life. Its approach has been sudden, and its termination often fatal in a very few days. Several infants have died of it, and even some advanced in life, notwithstanding the most active means that could be used.

Rheumatism, both acute and chronic, is still very common, even more so than last month. For the removal of acute rheumatism, bleeding, where necessary, with sudorific medicines and friction, have been generally successful. In my last Report, I mentioned the successful application of mercury in various forms, and the internal administration of arsenic. The last of these substances, however, cannot, from its activity, be with safety used on all occasions; and as I have found the following pills of great service in chronic rheumatism (a circumstance which I omitted in last report) I should recommend them, at least, previous to the exhibition of arsenic.

℞. Sulphur. antimonii, ppt. Submuriatis hydrarg. aa. gr. xxiv. Pulv. resin guaiac. gr. xlviii.

M. bene dein adde tinct. opii. q. s. ut fiat mass. quam divide in pil. xxiv.

Sig. Two pills to be taken at bed-time, and one in the morning.

Bowel complaints have been very common, and the young seem to have suffered in a greater proportion than those more advanced in life. This may probably be owing, in some degree, to the dampness of the weather; but I believe it is principally to be attributed to the immoderate use of fruits, especially of those not sufficiently ripe.

So violent have some of these cases been, that I have known a stout healthy child, for instance, reduced in two days in a most extraordinary manner; indeed, so much so, as even to alarm the relations with the fear of immediate danger.

Under this complaint various remedies were had recourse to, often coarse enough in their composition. Garlic and brandy seemed to be a favourite remedy among the good old wise matrons, and often before they would allow that their favourite remedy was ineffectual, the disease had frequently proceeded to an alarming state of severity.

Conceiving that the majority of these cases had their origin origin in the improper use of fruit, I have, in almost every instance, found, that the first and most effectual remedy was the administration of a smart emetic, after which a smart dose of any kind of physic: these, in the generality of cases, entirely removed the bowel complaint. In others, however, I have found it necessary to add to these the warm bath. This seemed to accelerate the evacuation of black hardened fæces, which being previously lodged in the intestines, probably gave origin to the original complaint. In one or two instances, there was, accompanying this complaint, a considerable degree of inflammation in the bowels. Under these circumstances, I have found a large blister, applied over the abdomen, to have the most beneficial effects.

*Princes Street.*

